# Therapeutics designed to neutralize soluble HIV tat protein could preserve IL-7 signaling and CD8 T-cell function in HIV+ patients

**DOI:** 10.1186/1758-2652-13-S4-P227

**Published:** 2010-11-08

**Authors:** EM Faller, SM Sugden, PA MacPherson

**Affiliations:** 1 Ottawa Hospital Research Institute, Ottawa, Canada

## Background

Interleukin (IL)-7 signaling is essential to CD8 T-cell development, homeostasis and function, and we have previously shown decreased expression of the IL-7 receptor alpha-chain (CD127) on CD8 T-cells in HIV+ patients. We have also shown that this down regulation of CD127 is mediated in part by soluble HIV Tat protein. By removing the IL-7 receptor from the cell surface, Tat is able to inhibit IL-7 signaling and impair both CD8 T-cell proliferation and cytolytic capacity.

## Purpose of the study

To determine the molecular mechanism by which Tat down regulates CD127.

## Methods

Histidine-tagged mutant Tat proteins were generated by sequentially deleting each of Tat's six domains and purifying the proteins over Nickel columns. CD8 T-cells were isolated from healthy HIV-negative volunteers and incubated in media alone or with purified Tat protein. CD127 surface and intracellular expression were measured by flow cytometry, fluorescence microscopy and by Western blot.

## Summary of results

Soluble Tat protein is taken up from the medium by CD8 T-cells via endocytosis. Once inside the cell, Tat exits the endosomes during their normal acidification, enters the cytosol, and then translocates to the inner leaflet of the cell membrane where it binds directly to the cytoplasmic tail of CD127. Tat then induces receptor aggregation and internalization through a process dependent on microtubules and directs CD127 to the proteasome for degradation. While the basic domain of Tat is required for entry into the cell, the N-terminal domain of Tat plays a key role in removing CD127 from the cell membrane. Anti-Tat antibodies, heparin, and colchicine all block Tat’s ability to down regulate CD127 on the cell surface.

## Conclusions

Given the important role of IL-7 in CD8 T-cell function, down regulation of the IL-7 receptor alpha-chain by Tat likely contributes to the impaired cell mediated immunity and inefficient immunologic control of viral replication evident in HIV+ patients with progressive disease. This makes Tat an attractive target for the development of new therapeutics and vaccines. Drugs designed to disrupt the interaction between Tat and CD127, or neutralizing anti-Tat antibodies induced by vaccination could restore CD127 expression and thus preserve CD8 T-cell function.

